# Spatial, Temporal, and Dietary Variables Associated with Elevated Mercury Exposure in Peruvian Riverine Communities Upstream and Downstream of Artisanal and Small-Scale Gold Mining

**DOI:** 10.3390/ijerph14121582

**Published:** 2017-12-15

**Authors:** Lauren Wyatt, Ernesto J. Ortiz, Beth Feingold, Axel Berky, Sarah Diringer, Ana Maria Morales, Elvis Rojas Jurado, Heileen Hsu-Kim, William Pan

**Affiliations:** 1 Nicholas School of the Environment, Duke University, Durham, NC 27710, USA; lauren.h.wyatt@duke.edu; 2 Global Health Institute, Duke University, Durham, NC 27710, USA; ernesto.ortiz@duke.edu (E.J.O.); axel.berky@duke.edu (A.B.); 3 University at Albany School of Public Health, State University of New York, Rensselaer, NY 12144, USA; bfeingold@albany.edu; 4 Pacific Institute, Oakland, CA 94612, USA; sdiringer@pacinst.org; 5 CENSAP, Puerto Maldonado, Madre de Dios 17000, Peru; anamorales30@hotmail.com; 6 Dirección Regional de Salud de Madre de Dios, Puerto Maldonado, Madre de Dios 17000, Peru; elvisrojasju@gmail.com; 7 Department of Civil and Environmental Engineering, Duke University, Durham, NC 27708, USA; hsukim@duke.edu

**Keywords:** mercury, diet, fish, exposure, temporal, ASGM, Madre de Dios, Peruvian Amazon

## Abstract

Artisanal and small-scale gold mining (ASGM) is a primary contributor to global mercury and its rapid expansion raises concern for human exposure. Non-occupational exposure risks are presumed to be strongly tied to environmental contamination; however, the relationship between environmental and human mercury exposure, how exposure has changed over time, and risk factors beyond fish consumption are not well understood in ASGM settings. In Peruvian riverine communities (*n* = 12), where ASGM has increased 4–6 fold over the past decade, we provide a large-scale assessment of the connection between environmental and human mercury exposure by comparing total mercury contents in human hair (2-cm segment, *n* = 231) to locally caught fish tissue, analyzing temporal exposure in women of child bearing age (WCBA, 15–49 years, *n* = 46) over one year, and evaluating general mercury exposure risks including fish and non-fish dietary items through household surveys and linear mixed models. Calculations of an individual’s oral reference dose using the total mercury content in locally-sourced fish underestimated the observed mercury exposure for individuals in many communities. This discrepancy was particularly evident in communities upstream of ASGM, where mercury levels in river fish, water, and sediment measurements from a previous study were low, yet hair mercury was chronically elevated. Hair from 86% of individuals and 77% of children exceeded a USEPA (U.S. Environmental Protection Agency) provisional level (1.2 µg/g) that could result in child developmental impairment. Chronically elevated mercury exposure was observed in the temporal analysis in WCBA. If the most recent exposure exceeded the USEPA level, there was a 97% probability that the individual exceeded that level 8–10 months of the previous year. Frequent household consumption of some fruits (tomato, banana) and grains (quinoa) was significantly associated with 29–75% reductions in hair mercury. Collectively, these data demonstrate that communities located hundreds of kilometers from ASGM are vulnerable to chronically elevated mercury exposure. Furthermore, unexpected associations with fish mercury contents and non-fish dietary intake highlight the need for more in-depth analyses of exposure regimes to identify the most vulnerable populations and to establish potential interventions.

## 1. Introduction

Artisanal and small-scale gold mining (ASGM), which uses liquid elemental mercury in the gold extraction process, has emerged as the leading anthropogenic source of mercury emissions globally and accounts for 37% of human related atmospheric emissions and an additional 800 tons of mercury are released to land and water annually [1,2]. Mercury is also released into the environment by other processes that include soil release from natural erosion, as well deforestation related to agricultural practices [3,4,5]. Environmental contamination poses a human health risk due to the potential for mercury to be transformed into the highly toxic and bioavailable form, monomethylmercury (MeHg), which is responsible for the majority of dietary human exposures [6]. MeHg is a potent neurotoxin with recognized impacts on child cognitive development [7,8,9]. Despite knowledge of these health impacts, ASGM continues to expand and has increased 200–500% since 1998 in some hotspots in Southeast Asia, Africa, and the Amazon, in response to the global demand for gold [10,11,12,13,14,15,16]. This rapid expansion raises concern for adverse human health impacts from elevated and chronic exposure in nearby non-ASGM communities. 

Adverse health impacts result from both acute and chronic exposures; however, chronic exposures are a serious health concern because they are not only associated with the developmental, renal, and neurological impacts observed with acute exposure, but also with cardiovascular and immunological impairments [17,18,19,20,21,22]. Additionally, chronic exposures could induce these health impacts at lower doses compared to acute exposures [23]. In regions impacted by ASGM, chronic mercury exposure is often presumed from occupational exposure or dietary consumption [8,24,25,26,27]; however, the majority of these studies use cross-sectional assessments of mercury content in hair, urine, or blood and only assess mercury exposure at one time-point [28]. In fact, we are aware of only a handful of studies that evaluated the variation in mercury exposure over time in ASGM regions [29,30,31]. Two of these studies, Dolbec et al. (2001) and Lebel et al. (1997), evaluated exposure seasonality in communities impacted by ASGM by using a segmented hair analysis. Both studies observed seasonality in hair mercury content that was correlated with fish consumption and fish species availability during different seasons [29,30]. More studies of this nature are needed to appropriately define the temporal aspect of exposure risk.

Furthermore, to properly predict human health outcomes in regions impacted by ASGM, understanding mercury exposure routes is critical. Currently, assessments of human exposure risk factors have primarily focused on general fish consumption and spatial relation to mining activities; however, methodological constraints of previous work have limited broader interpretation of the data. For example, when considering dietary exposure, only in a few studies has the focus been on some specific species, in combination with consumption frequency. The focus on fish is understandable, given the strong association with mercury exposure; however, this focus has led to a paucity of knowledge concerning other potential dietary associations. In two studies that evaluated other food groups [29,31], only one reported their observations of an association of reduced hair mercury with certain tropical fruits [31]. This reduction in hair mercury may relate to decreased MeHg bioaccessibility observed in vitro with certain phytochemicals in tea extracts and grains [32,33]. Laboratory-based studies also indicate that reduced mercury exposures could associate with dietary items rich in certain antioxidants, as antioxidants such as glutathione (GSH) and N-acetylcysteine (NAC) have been associated with increased mercury elimination [34,35,36]. Dietary selenium is another factor postulated to reduce mercury exposure, but observed outcomes in laboratory and epidemiological studies have not been consistent [37,38,39,40]. These findings warrant the consideration of other phytochemical and antioxidant-rich dietary items at the population level. In addition to dietary considerations, the prediction of human exposure risk requires knowledge of the spatial extent of environmental mercury contamination. In studies measuring both mercury content in fish and human hair, elevated exposures compared to a USEPA (U.S. Environmental Protection Agency) benchmark dose (1.2 µg/g) have been observed near and downstream of ASGM [30,41,42,43]. However, these studies fall short at describing the spatial extent of ASGM-related contamination as their mercury measurements were only made at one or two locations. A more extensive survey that measures exposure at different distances and directions from mining is necessary to better describe the extent of environmental contamination and identify where the risk of exposure is high.

Building on previous work, this study provides the largest mercury exposure assessment to date in communities impacted by ASGM in the Peruvian Amazon. We focus on exposure risks related to diet, spatial location, and proximity to ASGM activities to elucidate the relationship between environmental and human mercury exposure, evaluate chronic human exposure, and identify important risk factors for exposure in a region where ASGM has expanded rapidly over the past decades, Madre de Dios (MDD), Peru. In MDD, the potential for human exposure has been reported in two studies that describe a range of human exposure in six non-randomly selected communities [44,45]. While these studies indicate that human exposure is possible in some ASGM and non-ASGM communities in MDD, they have significant limitations connecting mining to fish contamination and human exposure because they: (1) did not assess human exposure in a systematic manner where communities and households were randomly selected; (2) only addressed fish consumption generally, not taking into account the importance of trophic level; and (3) measured levels of fish contamination in aquaculture farms, which are not a predominant source of fish for most households and could be less impacted by ASGM. Our study addresses these gaps, connecting human exposure and ASGM-related contamination by building on our previously published environmental study relating human exposure at different distances from mining to mercury content in water, sediment, and river fish [46]. The human exposure assessment presented in this study was conducted in the same communities and in a similar timeframe as our environmental study, and therefore enabled for the first time a direct comparison between environmental and human exposure in a population-representative cohort of communities located along a 560 km segment of the MDD River.

The first objective of this study was to determine how well human exposure can be predicted from environmental mercury levels and residential proximity to ASGM. We hypothesized human exposure would be related to environmental levels: low exposure would occur upstream where there are minimal mining impacts, the highest exposures would be in communities closer to ASGM, and slightly lower exposure would be observed downstream of mining. Secondly, because regional ASGM has been expanding in MDD, we also evaluated chronic exposure that was measured using sequential hair samples from women of child-bearing age (WCBA). Temporal mercury exposure was examined in WCBA as they are a sensitive population whose exposure directly influences child health. The Peruvian Ministry of Health recognizes the potential for adverse health impacts from increased mercury exposure in the region and is interested in better understanding exposure risk factors to facilitate the reduction in total mercury body burden in MDD communities. Finally, we sought to evaluate intra-household correlations of mercury exposure among children, parents, and other family members and determine whether other dietary items, including high antioxidant and selenium-rich foods, and diversity in food consumption are positively or negatively associated with hair mercury level. While it is suspected that the consumption of high trophic level fish species is a risk for mercury exposure, limited scientific evidence exists to define what other dietary items may be positively or negatively associated with mercury exposure.

## 2. Materials and Methods

### 2.1. Study Background and Population

Madre de Dios (MDD) is one of 24 administrative regions of Peru and is located in the southeast part of the country, in the southwestern Amazon Basin. The region is characterized by rainy and dry seasons and contains native and non-native communities. MDD contains the smallest population in Peru (~137,000, 2015), yet has the highest in-migration rate, the highest rate of population growth (2.5% per year), and one of the highest rates of deforestation since 2000 [47,48]. Two major developments are shaping MDD: ASGM expansion and the completion of the Interoceanic Highway in 2012. ASGM has existed in MDD for decades; however, the rapid rise in gold prices since 2007 followed by the presence of a well-maintained highway has resulted in ASGM and associated forest clearing to increase 400% [11,16]. 

### 2.2. Study Design and Data Collection

**Figure 1:**
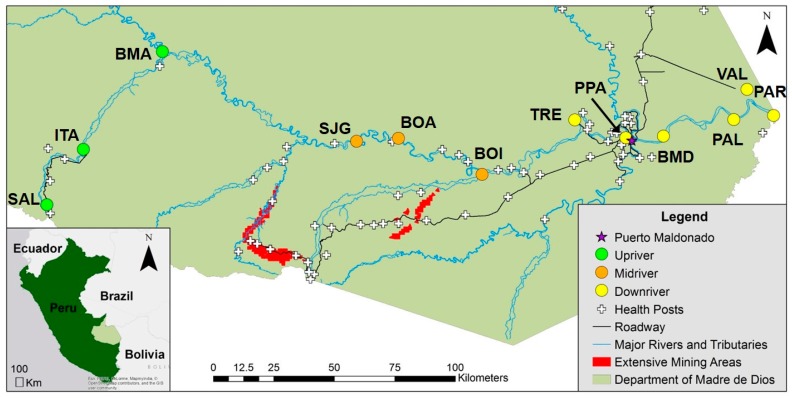
Map of selected study communities in Madre de Dios, Peru shown in reference to extensive mining areas. Study communities include: SAL, Salvacion; ITA, Itahuania; BMA, Boca Manu; SJG, San Juan Grande; BOA, Boca Amigo; BOI, Boca Inambari; TRE, Tres Islas; PPA, Puerto Pastora; BMD, Bajo Madre de Dios; PAL, Palma Real; PAR, Puerto Pardo; and VAL, Lago Valencia. Communities upstream of mining (SAL, ITA, and BMA) are indicated by dark green circles; communities near mining inputs (SJG, BOA, and BOI) are indicated by light green circles; and communities downstream of active mining (TRE, PPA, BMD, PAL, VAL, and PAR) are indicated by yellow circles.

This is a population-based study of native (indigenous) and non-native riverine communities where population livelihoods are strongly connected to the river through traditional land management and practices, including diet, occupation (fishing), and transportation [49,50,51,52,53,54]. A two-stage sampling procedure was implemented to select fifteen communities across four strata along the Rio Madre de Dios: (1) upstream of Rio Colorado; (2) between Rio Colorado and Rio Inambari; (3) Rio Inambari to Rio Tambopata; and (4) Rio Tambopata to the Bolivian border. Three communities were selected non-randomly: Boca Manu, Boca Colorado and Boca Inambari. The remaining 12 communities (Figure 1) were randomly selected by probability proportional to estimated size. Within each community, a minimum of four households were randomly selected to obtain a minimum of 20 participants per community. After consent was obtained, household surveys were administered by trained interviewers to obtain demographic, economic, nutritional (dietary), and migratory information (see below). Health data were also collected and included individual anthropometric measurements (height, weight, BMI, percent body fat, and percent body muscle). Percent body fat and muscle were estimated through bioelectrical impedance analysis using an OMRON HBF-514C portable body sensor. Stainless steel scissors and clippers were used to collect hair from the occipital region of the head and toenail samples, respectively, to test for metal exposures.

Due to social unrest at the time of initial data collection, communities were surveyed over two timeframes and three communities were not visited due to safety concerns. Communities upstream and downstream of intensive mining areas were enrolled between March and July 2014 and communities near active mining were enrolled in April 2016 (Figure 1). Overall, 278 participants were enrolled from 12 communities along the MDD River (Figure 1): three communities upstream of active mining (Salvacion, SAL; Itahuania, ITA; and Boca Manu, BMA); three communities close to mining (San Juan Grande, SJG; Boca Amigo, BOA; and Boca Inambari, BOI); and six communities downstream of active mining (Tres Islas, TRE; Puerto Pastora, PPA; Bajo Madre de Dios, BMD; Palma Real, PAL; Puerto Pardo, PAR; and Lago Valencia, VAL). The three communities not visited were considered unsafe or were flooded during both collection periods. Upstream communities were presumed beforehand to have less mercury exposure. This study was approved by the US Naval Medical Research Unit-6 Institutional review board and was supported by the Regional Health Directorate of Madre de Dios. All participants provided written informed consent and hair mercury results were shared with participants prior to publication.

Methods for sampling and measuring the mercury content in fish tissue were previously described by Diringer et al. (2015) [46]. Briefly, fish samples (*n* = 200) were acquired from local fishermen using traditional fishing techniques (123 carnivorous fish, 74 non-carnivorous fish). The total mercury in fish samples was determined by using a direct mercury analyzer that measures total mercury using direct thermal decomposition, amalgamation, and atomic absorption spectrometry (Milestone DMA-80) [46].

### 2.3. Household Food Consumption

Dietary information was collected at the household level, recording consumption (yes/no) and frequency (daily/weekly/sometimes/seasonally) of each food item. Household consumption was determined for: (1) antioxidant-rich items (quinoa, kiwicha, and tomatoes); (2) a selenium-rich item (Brazil nuts); (3) six commonly consumed fish (paco, bocachico, chambira, sabalo, doncella, and dorado) (Supplementary Materials, Table S1); (4) up to two additional fish (16 additional fish were indicated); and (5) additional non-fish items. Non-fish items included dietary variables in the following categories: cereals, tubers, vegetables, fruits, meat, eggs, nuts, dairy, and sweets (Supplementary Materials, Table S2). References to specific fish are based on common names, although scientific names were previously reported (Table S1) [46]. 

### 2.4. Hair Mercury Analysis

Mercury exposure for 231 individuals was estimated by measuring total mercury content in a proximal 2-cm segment of hair, corresponding with estimated exposure over the most recent two-month period before sample collection [6]. A lag of one month was used to adjust for the ~1 cm of hair retained in the scalp [55]. Total mercury was determined by direct combustion, gold amalgamation, atomic absorption spectrometry (Milestone DMA-80, Milestone SRL, Italy). The instrument calibration was verified by analysis of a hair standard reference material (ERM-DB001) every 10 samples in a batch run. Accepted measurements were within 10% of certified value. The detection limit was 1 ng Hg.

Temporal mercury exposure for WCBA (15–49 years, *n* = 46) was assessed by quantifying mercury contents of up to six 2-cm sequential segments to evaluate seasonal changes in exposure over 12 months. Variation over time was modeled using a cubic spline and by season (early-rainy, late-rainy, and dry seasons). The early-rainy season was considered as the period from December to March, late-rainy from April to July, and dry from August to November. Seasons were determined using national weather station data from Puerto Maldonado [56] and are confirmed by other work in the region [57,58]. The WCBA temporal subset includes the nine communities (SAL, ITA, BMA, TRE, PPA, BMD, PAL, VAL, and PAR) enrolled in 2014 to avoid potential exposure differences between the two collection timeframes.

### 2.5. Nail Selenium Analysis

Selenium exposure was estimated by measuring selenium content in toenails, which provide an estimate long-term exposure (4–6 months) [59,60,61]. Samples were scraped to remove visible dirt and then further cleaned with deionized water and HPLC-grade acetone. Samples were aggregated by individual and digested in trace metal grade hydrogen peroxide and nitric acid using the CEM Discover and Explorer SP-D microwave digestion system (CEM Corporation, Matthews, NC, USA). Selenium (Se) concentrations in the digestates were determined by inductively coupled plasma mass spectrometry (Agilent 7700). Quality control included method blanks to assess background contribution outside of the original sample, method controls to assess recovery without the matrix, and an ERM certified Reference Material to assess recovery of analytes in a similar matrix (human hair, ERM-DB001). The lower limit of detection was 0.085 ppb.

### 2.6. Statistical Analysis

Descriptive statistics using the geometric mean were used to describe the study population. Hair mercury content was compared to reference levels calculated from the USEPA benchmark dose for a maternal exposure level related to child developmental impairment (1.2 µg/g) and the level recognized by Peru’s government, which is calculated from the WHO provisional intake of methylmercury (2.0 µg/g) [6,62]. We report the proportion of individuals that exceed these levels and hair mercury content by sex and age. Differences in sex and age were evaluated using ANOVA and a generalized linear mixed model (GLMM) with a community random effect. Age was considered categorically with groups for adults (≥18 years) and children (<18 years). Children were also considered further with categories for young-adults (8–17 years) and younger children (<8 years). The distribution of hair mercury content was right skewed (Figure S1); thus, a log-transformation (log_10_) was used in subsequent analyses.

#### 2.6.1. Relationship between Environmental and Human Exposure

Community-level hair mercury was described using the geometric mean and evaluated using an unbalanced ANOVA. Communities were considered individually and in location based aggregates to determine the importance of location relative to mining and mining presence to hair mercury content. Location relative to mining was considered two ways: (1) with a binary variable for upstream (SAL, ITA, and BMA) and downstream (SJG, BOA, BOI, TRE, PPA, BMD, PAL, VAL, and PAR); and (2) with indicators for upstream (SAL, ITA, and BMA), active mining (SJG, BOA, BOI, and TRE), and downstream (PPA, BMD, PAL, VAL, and PAR). The importance of mining presence was determined with a binary variable for the presence (SJG, BOA, BOI, and TRE) and absence (SAL, ITA, BMA, PPA, BMD, PAL, VAL, and PAR) of mining within a community.

Pearson’s chi-square (goodness of fit) was used to test if the proportion of individuals (by community or area of the river) with hair mercury that exceeds the USEPA threshold (1.2 µg/g) deviates from the proportion that would be expected to exceed the USEPA oral reference dose based on environmental data and household consumption. The expected proportion was calculated from the fraction of individuals with a calculated oral reference dose that exceeded the USEPA reference dose of 0.1 µg/kg/day based on an individual’s weight, household fish consumption by trophic level, average mercury content in fish tissue, and an assumed portion size of 110 g (Oral reference dose calculation in Supplementary Materials) [62,63]. For each community or area of the river, mean fish tissue mercury by trophic level was calculated from fish within 100 km of that area to represent potential dietary exposure risk near the community. Fish species were classified by trophic level as in previous studies based on typical fish diet and included detritovores, herbivores, and filter feeders (trophic level 1); omnivores (trophic level 2); and obligate carnivores (trophic level 3) (Table S1) [46,64].

To further characterize the relationship between environmental and human exposure, and determine if human exposure was correlated with environmental exposures (fish source) in a certain direction of a community (nearby, upstream, or downstream) with respect to fish source, a GLMM was used to describe the relationship between human hair mercury and average mercury content in fish nearby (±50 km), upstream (±50 km of 50 km upstream), and downstream (±50 km of 50 km downstream). To control for the importance of fish consumption, an interaction between average fish mercury content and fish consumption by trophic level was included in the model. Average mercury content in fish for edge cases (upstream of SAL and ITA, downstream of PAL, PAR, and VAL) was imputed from a linear model predicting fish mercury content from a cubic consideration of location (distance from headwaters). Community was included as a random effect. 

#### 2.6.2. Temporal Variation in Hair Mercury

A GLMM was used to measure how well the mercury in the most recent hair segment predicted previous hair mercury content (log_10_). We calculated the odds and the probability that a hair segment exceeds one of the hair criterion if the most recent hair segment exceeded that criterion. GLMMs were also used to evaluate individual and household factors associated with hair mercury content as described in Section 2.6.3. GLMM models were adjusted for community location and partnership status, as being married or in a consensual union is associated with higher socioeconomic levels [65].

#### 2.6.3. Risk Factors Related to Hair Mercury

GLMMs were used to evaluate individual and household factors associated with hair mercury. GLMMs of continuous (log_10_) values of mercury were evaluated for three population subgroups: (1) the entire study population five years of age and older; (2) children under 10; and (3) WCBA. Dietary factors were analyzed separately for fish and non-fish items. Fish were analyzed by individual species and categorized by trophic level as in previous studies (Table S1) [46,64]. Non-fish items were analyzed by type and by computing dietary diversity using the Food and Agriculture Organization of the United Nations (FAO) dietary diversity guidelines [66]. A community’s location relative to mining (upstream, near, and downstream) and involvement in mining (active and not active) were evaluated as categorical variables. Household and community random effects were evaluated to adjust for correlated exposures within households and communities. Random intercepts for household and community were included in the model containing individuals five years or older. The child model included a random intercept for community alone. The WCBA model included a nested random effect (woman nested within community).

Prior to model building, a univariate analysis was used to screen variables that would be further considered in the multivariate models using a cut off of *p* < 0.2. Models were fit to determine the association of hypothesized factors to mercury levels in a sequential manner: fish consumption by trophic level and individually by species; demographic factors (age, sex); migration; location relative to active mining (present or absent in community); household factors (diet, average hair mercury in household, maternal hair mercury, paternal hair mercury, and average parental hair mercury); other food consumption; and nail selenium content. Variables included in the multivariate analysis were evaluated for multicollinearity using the variance inflation factor (VIF). Associations between child hair mercury content and the hair mercury content of their household members were evaluated using the average hair mercury of all household members, average hair mercury of parents, maternal hair mercury, and paternal hair mercury. Additionally, a GLMM was used to calculate the odds of a child exceeding 2.0 µg/g mercury if a household member also exceeded that level.

All analyses were conducted using R Version 3.4.0 (R Project; Vienna, Austria) and significance was evaluated at *p* < 0.05.

## 3. Results

### 3.1. Population Characteristics for Hair Mercury

Total hair mercury was elevated in Madre de Dios (MDD) (geometric mean: 2.6 µg/g 95% CI: 0.4, 10.5), with all communities having individuals who exceeded USEPA and WHO exposure limits (86% and 68%, respectively) (Table 1, Figure S1). Adults (≥18 years of age) had higher hair mercury compared to children (<18 years). Mercury levels did not differ by sex for adults (3.5 vs. 2.7 µg/g in males and females, respectively) nor children (1.9 vs. 2.5 µg/g in males and females, respectively). However, while average hair mercury content was similar between sexes, there were statistically significant age-sex differences (*p* = 0.009). Adult males had significantly higher hair mercury than male children (3.5 vs. 2.0 µg/g, *p* = 0.009), while females had similar mercury content between all age groups (2.5–2.7 µg/g) (Table 1, Figure S2). Of all children tested, 77% and 56% exceeded USEPA and Peruvian benchmarks for hair mercury contents, respectively, with the majority of girls exceeding both limits, 81% and 66%, respectively (Table 1).

### 3.2. Relationship between Environmental and Human Exposure Levels

**Figure 2:**
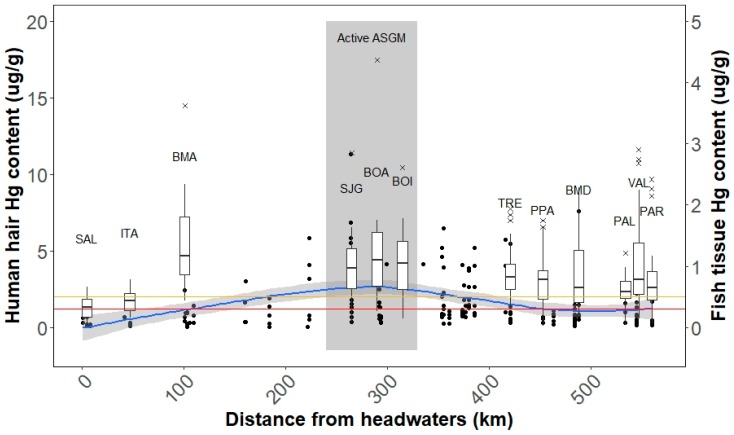
Total mercury content (µg/g) of human hair (left *y*-axis) and wet weight fish muscle (right *y*-axis) by location from headwaters along the Madre de Dios River. Boxplots indicate the variation in hair mercury and hair outliers are indicated by “x” symbols. Mercury content in fish tissue was reported previously and is indicated with black circles [46]. A LOESS (locally weighted smoothing) curve (blue line with grey 95% confidence intervals) was added to visualize the relationship between fish tissue mercury for all trophic levels and location along the river. Hair mercury limits for the USEPA (1.2 µg/g, red line) and WHO (2.0 µg/g, yellow line) are also indicated. The grey region indicates communities where intensive active mining is present (240–330 km).

Mid-river communities (i.e., near active mining areas) had significantly higher hair mercury compared to upstream and downstream communities (4.2 vs. 2.9 and 3.3 µg/g respectively, *p* < 0.001 and *p* = 0.03 respectively); however, among individual communities, the highest and lowest average hair mercury content was detected in upstream communities where environmental mercury levels were low (Table 1 and Figure 2): BMA with an average hair mercury content of 4.8 µg/g (95% CI: 1.8, 11.7) and SAL with an average of 1.0 µg/g (95% CI: 0.2, 2.6). Communities with an active mining presence also had significantly higher hair mercury than communities with no mining presence (4.2 vs. 3.2 µg/g respectively, *p* = 0.001). Communities in active mining areas and downstream had the highest prevalence of individuals exceeding 2.0 µg/g (80% and 62% of individuals respectively) (Table 1, Figure S3).

The lowest mercury concentrations in human hair and the previously described environmental sediment and fish samples were observed near headwaters (0–50 km) [46]. Of the referenced environmental samples, the greatest mercury content in fish tissue and sediment MeHg was observed 200–400 km downstream and slightly lower mercury concentrations were observed further downstream [46]. However, human samples exhibited a different spatial pattern (Figure 2). The greatest hair mercury was observed in BMA, a community more than 75 km upstream of mining, in a location where low environmental mercury contents (sediment and fish) were observed. Further downstream, hair mercury aligned with a similar spatial trend to the environmental samples, with high hair content between 200 and 400 km (SJG, BOA, and BOI) and slightly lower mercury content further downstream (TRE, PPA, BMD, PAL, PAR, and VAL) (Figure 2). 

We attempted to estimate potential mercury body burden using fish tissue mercury contents and household fish consumption, as outlined in Supplementary Materials. The calculation for each individual utilized an individual’s weight, household fish consumption by trophic level, mercury content in fish collected near an individual’s community, and estimated portion size for each fish meal. The results were used to estimate the proportion of individuals that would be expected to have hair mercury values that exceed the USEPA threshold and indicated that mercury hair levels were underestimated by an average of 26% across all communities and by 48% for BMA. This difference was significant when individuals were grouped by community and larger river segments (*p* < 0.001) (Figure S4).

When considered alone, mercury content in local fish was not a strong predictor of human mercury exposure as no relationship was observed between the three location variables (fish near, upstream, and downstream) and hair mercury content. Consumption of high trophic level fish was the best predictor for hair mercury content. Although the interactions between fish mercury content and household level fish consumption by trophic level were not statistically significant, the relationship between trophic level 3 consumption and higher hair mercury approached statistical significance (*p* = 0.06). 

### 3.3. Temporal Variation in Hair Mercury

The analysis of women’s hair to assess temporal mercury exposure revealed that hair mercury contents were continuously high and did not follow a seasonal pattern. The geometric mean of a WCBA’s hair segment average was 3.2 µg/g (95% CI: 0.4, 11.7) and the majority of hair segments exceeded 1.2 µg/g: 96% had at least one hair segment above this level and 80% had all segments above this level. If the most recent 2-cm hair segment exceeded the 1.2 µg/g reference value, there was a 97.4% probability that hair mercury exceeded this value in the past 8–10 months. In addition to a consistent mean, hair mercury values did not vary over time as 63% of hair mercury segments were within 20% of the most proximal 2-cm hair segment and only 26% were below the 2-cm value. Remarkably elevated hair mercury was observed in several women (7) who had hair mercury above 10 µg/g for at least 2 sequential hair segments, representing 4 months (Figure S5). 

The WCBA model to assess risk factors was adjusted for community location and partnership status, to control for socio-economic status. Significant positive associations were observed between hair mercury and high general fish consumption and at least weekly consumption of trophic level 3 fish (doncella and chambira) (Figure S6, Table S3). All WCBA resided in a household that consumed fish and 63% resided in a household that consumed fish at least weekly (Table 2). 

**Figure 3:**
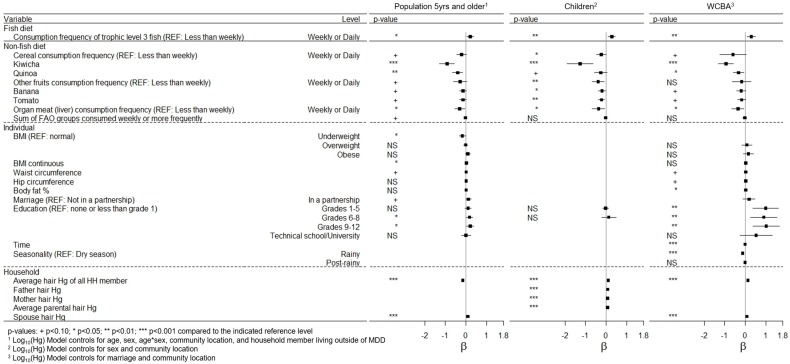
Association between hair mercury and dietary, individual, and household factors in the population five years and older, children, and women of child bearing age (WCBA).

**Figure 4:**
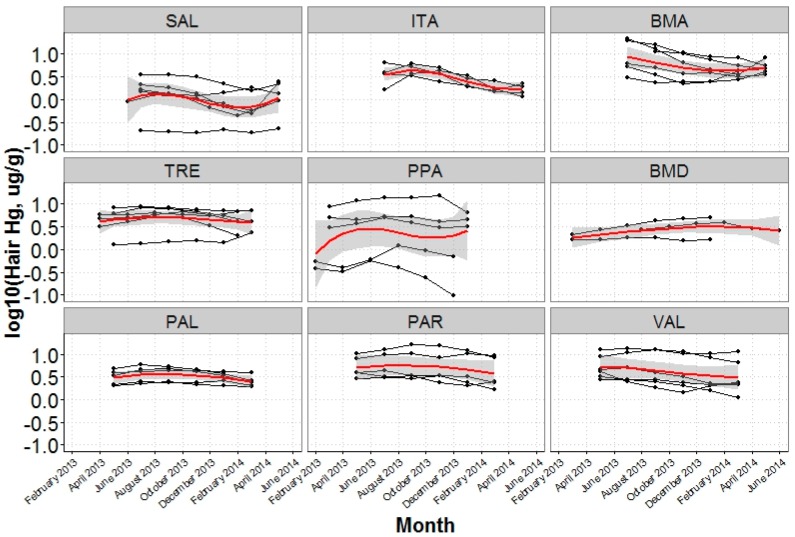
Temporal mercury exposure over a year timeframe was measured in women of child bearing age (WCBA) (*n* = 46). Differences by community are depicted with black lines connecting hair mercury contents (log_10_) of each individual woman over 2-cm (approximately 2 month) intervals. The modeled relationship with cubic splines is indicated by the red line with the grey area indicating the 95% confidence interval. Rainy season was considered as the period from December to March, post-rainy season from April to July, and dry season from August to November. Upstream communities (SAL, ITA, and BMA) are indicated in the top row and downstream communities (TRE, PPA, BMD, PAL, VAL, and PAR) in the middle and bottom rows.

Models to assess household non-fish dietary items, and household and individual variables were also adjusted for the household consumption of trophic level 3 fish. Similar to the fish variables, significantly reduced hair mercury was observed with frequent kiwicha, quinoa, bananas, tomatoes, and liver consumption at the household level. Household dietary diversity was not significant in the WCBA model when hair mercury beyond the most recent 2 months was considered (Figure 3 and Figure S5). Significant seasonality with hair mercury was observed where lower hair mercury content was associated with the rainy season (28% reduction compared to the dry season) (Figure 3 and Figure 4, and Figure S5). Hair mercury was also significantly positively related to body fat percent (*β* = 0.01, 95% CI: 0.00, 0.02) (Figure 3, Table S4).

### 3.4. Risk Factors Related to Hair Mercury

Significant associations were observed with household member hair mercury. In the model with individuals over five years, average household hair mercury was negatively associated with an individual’s hair mercury (*β* = −0.2, 95% CI: −0.2, −0.1), likely related to males >40 years having higher hair mercury content as this association was reversed when older males were removed from the model (*β* = 0.1, 95% CI: 0.08, 0.12). However, child hair mercury content was significantly and positively associated with the average hair mercury content of parents and other household members (Figure 3, Table S3). Children had 2.4 times higher odds of their hair mercury exceeding 1.2 µg/g (95% CI 1.9–3.1) if their mother’s hair mercury exceeded this level, compared to a child whose mother did not exceed the level in a univariate model (Table 3). Additionally, an individual had 1.9 times higher odds of their hair mercury exceeding 1.2 µg/g (95% CI 1.2–2.0) if the WCBA in their household had hair that exceeded this level (*p* < 0.001). In the temporal analysis, WCBA were observed to have hair mercury elevated over the previous year, which suggests that exposure is chronically elevated in all household members. There was a small but significant relationship between body mass and hair mercury, where being underweight was associated with lower hair mercury in the model for individuals over five years (*β* = −0.2, 95% CI: −0.3, −0.0) (Figure 3, Table S3). For a subset of individuals with nail Se data, a statistically significant association between log_10_Se and logged hair mercury content was not observed.

Linear models for children, individuals five years and older, and WCBA consistently demonstrated that higher hair mercury is associated with more fish consumption, particularly weekly or more frequent consumption of high trophic level fish. When considering fish diet variables in both models, hair mercury was significantly positively associated with high general fish consumption and at least weekly consumption of trophic level 3 fish, including doncella and chambira. In the three population models, hair mercury was significantly increased by 92–99% with high general fish consumption (compared to low consumption), 61–84% with at least weekly consumption of trophic level 3 fish (compared to less than weekly consumption), 79–109% with weekly or daily doncella consumption (compared to less than weekly consumption), 57–76% with weekly or daily chambira consumption (compared to less than weekly consumption), and 87–155% with weekly or daily yulilla (trophic level 1) consumption (compared to less than weekly consumption) (Figure S5, Table S3). Fish are an important protein source to riverine MDD communities with 97% of households reporting fish consumption and half of these households consuming fish at least weekly (Table 2). The majority of households (73%) consume fish from the river, but other sources of fish that are consumed include lakes (21%), markets (19%), and fish farms (2%). Weekly or more frequent fish consumption was common in upstream and downstream communities; however, no households near active mining reported weekly or daily fish consumption. Households in communities near active mining all reported consuming fish “sometimes”.

In the models to assess non-fish dietary variables, as well as household and individual factors, the consumption of trophic level 3 fish was additionally included as a covariate. In both models, frequent consumption of cereals, fruits, and liver were significantly and negatively associated with hair mercury. Kiwicha, quinoa, bananas, tomatoes, and liver were associated with 88–95, 46–61, 29–38, 29–41, and 50–58% reductions in hair mercury, respectively, with weekly or more frequent consumption. Frequent consumption (weekly or daily) of antioxidant-rich items occurred in 28 of the 59 total households, though fewer households reported frequent kiwicha and quinoa consumption, 2 and 6 household respectively (Table 2). Weekly or daily consumption of the selenium-rich item (Brazil nuts) was reported in 4 households and was not significantly associated with hair mercury (Figure 3, Table 2). In the model with all individuals over five years, increasing dietary diversity was significantly associated with an 8% reduction in hair mercury. Dietary diversity had a near significant reduction in hair mercury in children (*p* = 0.13).

## 4. Discussion

This is the first study to provide a spatially-resolved assessment of local environment, diet, and mercury exposure across a large geographic region of substantial ASGM activity with a large study population. Results from our studies demonstrate extensive environmental mercury contamination near and far downstream of ASGM activities are directly correlated with elevated human exposure across all age-sex groups. In addition, we identify a population far upstream (more than 100 km) of mining where environmental mercury contamination is low, but human mercury exposure is high. Lastly, we have determined that temporal variability in mercury exposure is low, yet considerable variability in mean levels exist across communities. The disparity between low environmental mercury and elevated human exposure persisted after accounting for household fish consumption and indicates that environmental sampling alone is insufficient to evaluate human mercury exposure. Fish consumption was strongly associated with increased hair mercury content; however, this study also observed lower hair mercury associations with frequent fruit and grain consumption. Although directionality of the inverse associations could not be addressed in this cross-sectional study, these results provide additional support that certain nutrients, such as omega-3 fatty acids [67,68], have an influence on mediating mercury exposure and toxicity, which may account for the conflicting results concerning mercury related child neurotoxicity in two prospective studies performed in the Faroe Islands, where neurotoxicity was observed, and the Seychelles, where significant neurotoxicity was not observed. 

Mercury levels detected among residents living in the MDD watershed were elevated over the year timeframe assessed in this study. The observed levels were greater than those reported in a previous study in Puerto Maldonado [44], but lower than other studies of high fish-consuming communities of the Brazilian Amazon [69,70,71]. In Brazil, along the Rio Tapajós, observed mercury content in hair ranges 4–9 µg/g in areas not impacted by mining [72] and 13–24 µg/g in areas impacted by gold mining [71,73]. In MDD, more than 80% of WCBA exceeded the USEPA threshold of 1.2 µg/g at the time of sampling and for the majority of the prior year. These results, combined with the model of child-predicted hair mercury imply that children who currently exceed 1.2 µg/g have an 85% probability of exceeding 1.2 µg/g for 8 of the past 12 months. These exposure levels have been associated with neurological deficits including child cognition and visual recognition, as well as verbal performance, visual recognition, language, attention, and memory deficiencies later in childhood in multiple longitudinal studies [7,74] and a cross-sectional study in an Amazonian population [8]. In MDD, the estimated neurotoxicity effects could include reduced infant visual recognition memory (VRM) score by 19.9 points (up to 70.9) and child IQ by 1.2 points (up to 4.4) based on reduced visual recognition memory scores in US infants [74] and an estimated IQ point reduction from an assumed linear dose-response from neuropsychological exams administered to the Faroe Islands study cohort at age 7 [75,76]. Moreover, a more recent evaluation of the Faroe Island cohort at age 22 years provides evidence that these deficits may persist beyond childhood [77]. Child exposure and developmental outcomes are areas that should be pursued further as mercury exposure in these communities is chronic, which could produce long-term disease risk and loss in economic productivity [78].

Over the timeframe evaluated, we observed high exposure over the previous year throughout all study communities, though temporal exposure varied between individual WCBA. In addition, we observed exposure seasonality, with higher hair mercury during the dry season and lower hair mercury during the rainy season. Although we were unable to assess seasonal availability of fish in each community, the lower exposure during the rainy season and could be reflective of an overall reduced fish availability or altered species availability, as mercury content in fish has not been observed to vary between the wet and dry seasons [46]. A comparable pattern of lower exposures around the February–March rainy season was observed in another Amazonian population that had increased herbivorous fish consumption during the period of lower hair mercury, although the seasonality was more pronounced in that study [29]. Interestingly, our observed changes in hair mercury over time did not show evidence of a continual increase in mercury exposure that could be expected in a region where mining has been readily expanding. However, the lack of an apparent increase in hair mercury may relate to the short timeframe examined. 

The patterns in exposure over time varied among women, though the range between the maximum and minimum hair mercury for most women was modest (less than 5 µg/g). This was not the case for all women, as a larger range (of up to 17 µg/g) was noted in several women with the highest exposures. The highest hair mercury contents (above 10 µg/g) were recorded during July–November 2013 in three Boca Manu (BMA) women with similar temporal exposure patterns. Hair mercury content in these individuals rapidly decreased to a lower level (5 µg/g) over 6–8 months. This attenuation of hair mercury was similar to the log-linear elimination rate observed in animal and human models recorded following a single exposure [79]. All three women were from households that reported seasonal fish consumption and our observations could relate to attenuated mercury exposure following a high dietary exposure event related to season.

Spatial location relative to mining was an important determinant of both environmental and human mercury exposure, with the lowest exposures occurring more than 100 km upstream from ASGM and increased exposures occurring in near and far downstream of active mining. The correlation between human exposure and our environmental data that was collected in a similar timeframe with human biomarkers was high for communities near and downstream of ASGM. However, our results suggest that environmental mercury level (fish contamination) is not an adequate predictor in some communities. For example, communities near mining may have unexpected dietary sources or be exposed to other mercury species. Because the mercury in hair is typically dominated by monomethylmercury species [80], diet is often presumed to be the dominant exposure route; however, a fraction of mercury in hair could be the inorganic form caused by other dietary and non-dietary routes (e.g., inorganic mercury exposure from gold amalgamation) [81]. Unexpectedly, the highest measurements of hair mercury were identified in Boca Manu, a community located ~75 km upstream of active mining (Figure 2). In communities upstream of mining, the high human exposure may relate to migration patterns to areas with higher mercury exposure risk, an unmeasured genetics factor causing slower clearance of mercury, or a difference in natural geology that results in increased local mercury release. These observed variations in predicted mercury levels underscore the importance of evaluating more information to predict human exposure.

In accordance with other studies, fish consumption was a strong predictor of increased mercury exposure [82,83,84,85]. While general fish consumption at the household level did not predict hair mercury content, the consumption of higher trophic level fish including doncella and chambira, was associated with a 57–168% increase in hair mercury. Environmental data from the region also support that these fish could pose a risk as the average fish filet mercury level of doncella and carnivorous fish was above the USEPA recommended value (0.3 mg/kg), with many fish above the higher United States Food and Drug Administration (USFDA) action level (1.0 mg/kg) [46].

Other household dietary variables were found to be strong predictors of mercury exposure, including the frequent consumption of grains and fruits native to Peru, which were associated with decreased hair mercury. The influence of two grains, quinoa and kiwicha, on mercury exposure have not been previously examined and frequent household consumption of these grains was associated with 61–95% and 45–88% lower hair mercury contents, respectively. The correlation of frequent grain consumption with decreased exposure may be related to increased mercury elimination. Both quinoa and kiwicha have health benefits that are suggested to originate from being high in fiber, dietary minerals (calcium, magnesium, manganese, and phosphorus), antioxidants, and flavonoids [86,87,88,89]. 

Reduced hair mercury was also correlated with frequent fruit consumption, and the strongest associations were observed with tomatoes and bananas. Increased tomato and banana consumption were associated with 29–41% reductions in hair mercury. Protective effects related to tomato consumption has been observed in other studies [90,91,92]. In young children, tomato consumption has been associated with lower blood mercury levels [90]. In laboratory studies, tomato and tomato extracts mitigated the impacts of inorganic mercury exposure on intercellular communication and cytokine concentrations [92] and reduced mercury accumulation in liver [91]. Mechanisms related to this inverse relationship have not been identified but are likely related to the nutritional value of tomatoes and increased mercury elimination as tomatoes are known to contain antioxidants (the carotenoid lycopene, ascorbic acid, and beta-carotene); flavonoids; and metal chelating proteins, peptides, phytochelatins, and other heavy metal binding complexes that are analogous to metallothioneins [93,94,95]. Mechanisms for mercury reductions observed with banana consumption are likely similar to that of tomatoes, as bananas also have high levels of antioxidants (*β*-carotene, vitamin C, and vitamin E) [96,97]. Our observations of reduced mercury exposure associated with consumption of certain dietary items should be further investigated with mechanistic studies and dietary interventions to validate these findings as they have the potential to mediate mercury exposure in communities where exposure is elevated. Considering diet as a factor that contributes to health status is not new; however, it is increasingly important to consider when determining mercury exposure impacts. Certain nutritional factors, like selenium, are hypothesized to alter mercury exposure by reducing bioavailable mercury and increasing antioxidant function [37,98,99,100,101,102], while others, such as omega-3 fatty acids and vitamin B, have been associated with modifying child neurological [67,68,74,103] and immunological outcomes [104,105].

## 5. Conclusions

In January 2016, Peru ratified the articles of the Minamata Convention Treaty, obligating the country to curb mercury emissions and monitor vulnerable populations at risk of exposure. The treaty became effective August 2017, with this study providing one of the first comprehensive evaluations of mercury exposure in mining and non-mining individuals as well as for women of child-bearing age. Our findings justify the immediate need for a biomonitoring program in Madre de Dios as well as in other regions of the world where ASGM activities are incipient. While we recognize that our sample size is modest, the persistent temporal nature of mercury exposure in a highly vulnerable population that is economically and spatially disconnected from ASGM is a major public health emergency. This study also identifies potential dietary interventions that may mitigate mercury absorption (quinoa, kiwicha, and some fruits). While considering diet at the household level may be appropriate for this population, future studies should assess individual dietary patterns and other potential mechanisms of exposure. Finally, these results support the need for additional research on health impacts following mercury exposure in ASGM regions, particularly in regions where fish consumption is high and health problems, particularly infectious diseases and malnutrition, could moderate mercury exposure levels.

## Figures and Tables

**Table 1:** Summary of hair mercury (µg/g) distribution by sex, age, and community.

Population Grouping		N	Geometric Mean Hair Hg (95% CI)	% Individuals above 1.2 µg/g ^a^	% Individuals above 2.0 µg/g ^b^
Total		231	2.6 (0.4, 10.5)	85.7	68.4
Sex and age in years					
Female	<8	32	2.5 (0.4, 9.4)	81.3	65.6
	8–17	26	2.4 (0.9, 6.3)	92.3	65.4
	18+	61	2.7 (0.2, 11.5)	85.2	68.9
Male	<8	25	1.9 (0.4, 4.8)	72.0	60.0
	8–17	31	1.9 (0.4, 5.2)	77.4	48.4
	18+	56	3.5 (1.2, 10.5)	96.4	85.7
Community					
Upstream	SAL	19	1.0 (0.2, 2.6)	52.6	26.3
	ITA	22	1.6 (0.7, 3.1)	68.2	31.8
	BMA	23	4.8 (1.8, 11.7)	100.0	91.3
Midriver (near mining)	SJG	13	3.2 (0.8, 9.9)	92.3	84.6
	BOA	14	4.1 (1.4, 14.1)	92.9	92.9
	BOI	10	3.5 (0.9, 9.7)	90.0	80.0
Downstream	TRE	23	3.3 (1.5, 7.5)	100.0	91.3
	PPA	19	2.2 (0.2, 6.8)	84.2	63.2
	BMD	21	2.8 (1.0, 8.5)	90.5	61.9
	PAL	25	2.1 (0.6, 4.3)	88.0	64.0
	PAR	20	2.6 (0.5, 9.4)	90.0	70.0
	VAL	22	3.1 (0.7, 11.3)	81.8	77.3

^a^ USEPA level (1.2 µg/g); ^b^ Peruvian government and WHO level (2.0 µg/g).

**Table 2:** Summary of hair mercury (µg/g) by household (HH) level consumption frequency of fish, antioxidant-rich (quinoa, kiwicha, tomato), and selenium-rich (Brazil nuts) diet items.

Consumption Frequency		HH (N)	People (N)	Geometric Mean Hg All HH Individuals (95% CI)	Geometric Mean Hg WCBA (95% CI)	Geometric Mean Hg Children <10 Years (95% CI)
Fish	Never/Seasonally/Sometimes	30	113	2.9 (0.4, 9.6)	2.7 (0.4, 7.7)	2.2 (0.3, 6.7)
	Weekly	11	45	1.7 (0.3, 4.6)	1.5 (0.3, 4.5)	1.6 (0.8, 3.9)
	Daily	18	73	2.9 (0.6, 11.1)	3.4 (1.3, 10.6)	2.3 (0.4, 9.7)
Antioxidant-rich	Never/Seasonally/Sometimes	31	120	3.0 (0.6, 11.0)	2.9 (1.1, 10.8)	2.6 (0.3, 8.9)
	Weekly	6	23	2.1 (0.7, 8.4)	2.3 (0.9, 7.5)	1.6 (0.6, 4.2)
	Daily	22	88	2.2 (0.2, 8.8)	2.4 (0.2, 7.8)	1.6 (0.3, 6.3)
Selenium-rich	Never/Seasonally/Sometimes	55	215	2.6 (0.3, 10.6)	2.5 (0.2, 9.0)	2.1 (0.2, 8.7)
	Weekly	1	3	2.1 (0.9, 5.1)	2.1 (2.1, 2.1)	0.8 (0.8, 0.8)
	Daily	3	13	2.4 (0.8, 7.3)	3.3 (1.5, 8.3)	2.6 (2.2, 3.0)

**Table 3:** Odds ratio (OR) for a child (<10 years) exceeding the 1.2 µg/g level based on family and sex variables.

Variable	Family and Sex Variables	Odds Ratio (OR)	95% CI
Family	Paternal hair Hg exceeds level	2.14	(1.17, 3.93) ^†^
	Maternal hair Hg exceeds level	2.39	(1.86, 3.07) ^†††^
	Parent hair Hg exceeds level	2.19	(1.24, 3.90) ^††^
	Mean HH hair Hg exceeds level	2.32	(1.73, 3.12) ^†††^
	Mean HH adult hair Hg exceeds level	2.29	(1.54, 3.38) ^†††^
Sex	Female	1.12	(0.92, 1.36)

^†^ *p* < 0.05; ^††^ *p* < 0.01; ^†††^ *p* < 0.001 compared to a child not exceeding the level.

## Supplementary Materials

The following are available online at www.mdpi.com/1660-4601/14/12/1582/s1, Figure S1: Distribution of hair mercury in the study population (*n* = 231). Eighty-four percent of individuals had hair mercury contents above the USEPA limit (1.2 µg/g, dashed red line) and 65% had levels above the WHO limit (2.0 µg/g, dashed yellow line). Skewness values by community: SAL 0.30, ITA 0.30, BMA 1.27, SJG 1.35, BOA 2.21, BOI 0.70, TRE 0.97, PPA 0.60, BMD 0.85, PAL 0.15, VAL 1.05, and PAR 1.36. Kurtosis values by community: SAL 1.90, ITA 2.02, BMA 4.51, SJG 5.00, BOA 7.74, BOI 2.91, TRE 3.01, PPA 2.61, BMD 2.69, PAL 3.02, VAL 1.05, and PAR 3.78, Figure S2: The relationship between hair mercury content and age varies by sex. This relationship is represented with a linear relationship (red line with the 95% confidence interval indicated in grey) and one with cubic splines (blue line with the 95% confidence interval indicated in grey). Horizontally, the USEPA limit (1.2 µg/g, red line) and WHO limit (2.0 µg/g, yellow line) are indicated, Figure S3: Hair mercury contents varied by community with many individuals having levels above the USEPA limit (1.2 µg/g, red line) and WHO limit (2.0 µg/g, yellow line). The grey region indicates communities where intensive active mining is present, Figure S4: (Panel A) Average percent difference (±SE) of HH members that were observed to exceed the USEPA limit (1.2 μg/g) compared to the expected proportion that would exceed the limit based on the proportion of fish caught near (±50 km) the community that exceeded the USEPA limit for water quality (0.3 mg/kg). HHs are grouped by location relative to mining (upstream, near active mining, and downstream) and by carnivorous fish consumption (less than weekly, weekly or greater consumption). (Panel B) The proportion of observed individuals from community groupings that had hair mercury content that exceeded the USEPA limit compared to the expected proportion that would exceed the limit based on the proportion of fish caught near (±50 km) the community that exceeded the USEPA limit for water quality. Community groupings are as follows A: communities upstream of mining with low hair mercury (SAL, ITA), B: communities upstream and high hair mercury (BMA), C: communities near mining inputs (SJG, BOA, BOI), D: communities downstream that are downstream of mining (TRE, PPA), and E: communities further downstream (BMD, PAL, PAR, VAL), Figure S5: Temporal exposure over a year timeframe was measured in WCBA (*n* = 46). Differences by community are depicted with lines connecting hair mercury contents (ppm) of each individual woman over 2-cm (approximately 2 month) intervals. The red line representing the USEPA limit (1.2 µg/g, dashed red line) and WHO limit (2.0 µg/g, dashed yellow line), Figure S6: Association between hair mercury and dietary fish consumption in the population five years and older, children, and WCBA, Table S1: Trophic levels of fish, Table S2: FAO food categories for non-fish items, Table S3: Association between hair mercury and dietary, individual, and household factors in the population five years of age and older, WCBA, and children, Table S4: Association between hair mercury and fish consumption in the population five years of age and older, WCBA, and children.
