# Supplementary material for: Spatial, Temporal, and Dietary Variables Associated with Elevated Mercury Exposure in Peruvian Riverine Communities Upstream and Downstream of Artisanal and Small-Scale Gold Mining
Source: Int J Environ Res Public Health. 2017 Dec 15;14(12):1582. doi: 10.3390/ijerph14121582 (PMC5751000; doi:10.3390/ijerph14121582)
Supplement: Supplementary file 1 [file ijerph-14-01582-s001.pdf]

Supplementary Materials

Oral reference dose calculation

The proportion of individuals with an oral reference dose that exceeded the USEPA reference dose of 0.1 µg/kg/day was calculated using the weight of an individual (kg), household fish consumption by trophic level, average mercury content in fish tissue, and an assumed portion size of 110 g [1,2]. Household fish consumption was estimated from survey responses based on fish type and trophic level (Table S1). The sum yearly consumption for fish by trophic level was calculated based on survey responses that included daily (365 d), weekly (52 d), seasonally or sometimes (13 d), and never (0 d). This yearly total was divided by 365 to calculate daily intake (times/d). The mean mercury content of fish (mg/kg) by trophic level was calculated from fish near (100 km upstream and downstream) each community or area of the river. The following equation was utilized to calculate an individual's oral reference dose (mg/kg/day):

$$[(\text{mean fish Hg content}_{(\text{trophic level 1})} \times \text{portion size} \times \text{daily intake}_{(\text{trophic level 1})} \div \text{weight}] +$$
$$[(\text{mean fish Hg content}_{(\text{trophic level 2})} \times \text{portion size} \times \text{daily intake}_{(\text{trophic level 2})} \div \text{weight}] +$$
$$[(\text{mean fish Hg content}_{(\text{trophic level 3})} \times \text{portion size} \times \text{daily intake}_{(\text{trophic level 3})} \div \text{weight}].$$

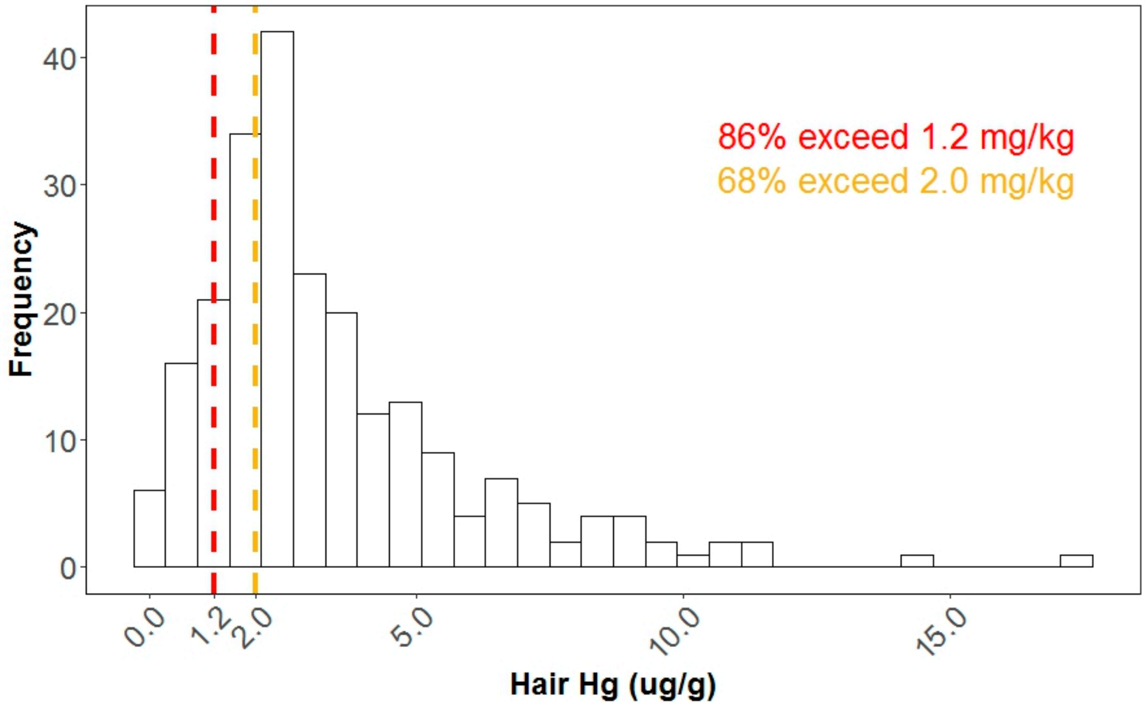

Figure S1. Distribution of hair mercury in the study population (*n* = 231). 84% of individuals had hair mercury contents above the USEPA limit (1.2 µg/g, dashed red line) and 65% had levels above the WHO limit (2.0 µg/g, dashed yellow line). Skewness values by community: SAL 0.30, ITA 0.30, BMA 1.27, SJG 1.35, BOA 2.21, BOI 0.70, TRE 0.97, PPA 0.60, BMD 0.85, PAL 0.15, VAL 1.05, and PAR 1.36. Kurtosis values by community: SAL 1.90, ITA 2.02, BMA 4.51, SJG 5.00, BOA 7.74, BOI 2.91, TRE 3.01, PPA 2.61, BMD 2.69, PAL 3.02, VAL 1.05, and PAR 3.78.

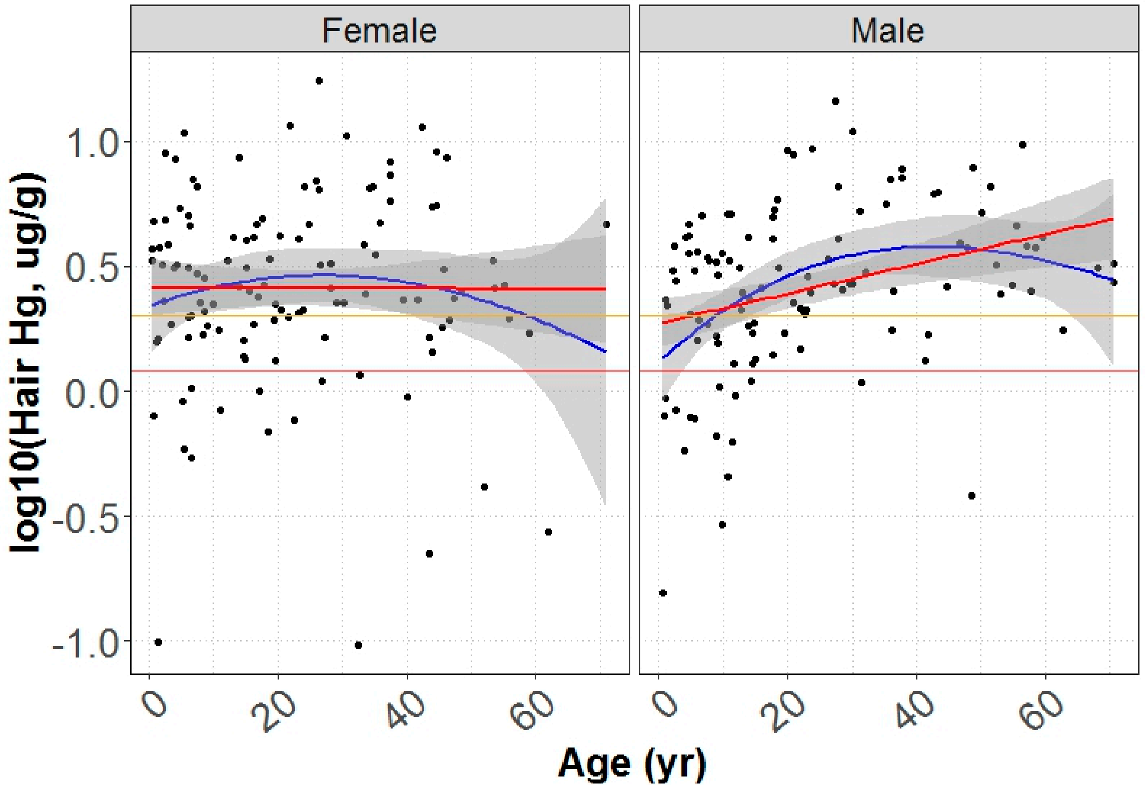

Figure S2. The relationship between hair mercury content and age varies by sex. This relationship is represented with a linear relationship (red line with the 95% confidence interval indicated in grey) and one with cubic splines (blue line with the 95% confidence interval indicated in grey). Horizontally, the USEPA limit (1.2 μg/g, red line) and WHO limit (2.0 μg/g, yellow line) are indicated.

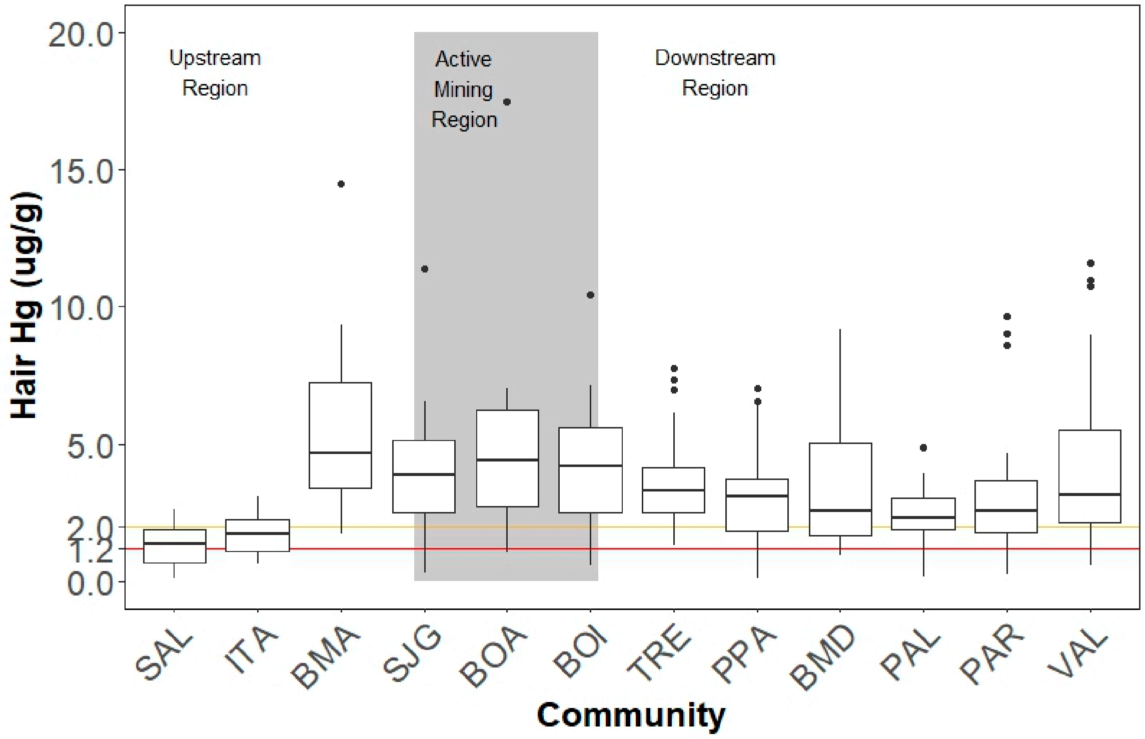

Figure S3. Hair mercury contents varied by community with many individuals having levels above the USEPA limit (1.2 μg/g, red line) and WHO limit (2.0 μg/g, yellow line). The grey region indicates communities where intensive active mining is present.

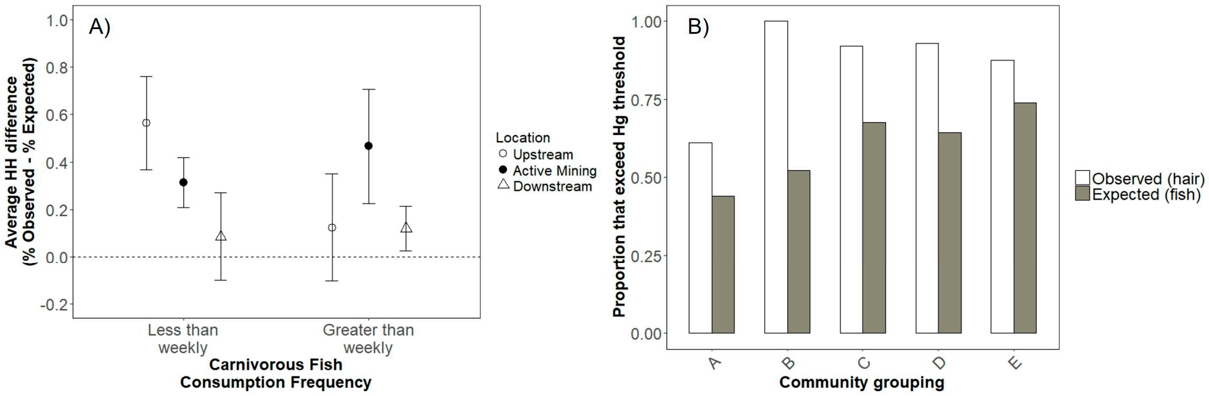

Figure S4. (Panel A) Average percent difference (±SE) of HH members that were observed to exceed the USEPA limit (1.2 μg/g) compared to the expected proportion that would exceed the limit based on the proportion of fish caught near (±50 km) the community that exceeded the USEPA limit for water quality (0.3 mg/kg). HHs are grouped by location relative to mining (upstream, near active mining, and downstream) and by carnivorous fish consumption (less than weekly, weekly or greater consumption). (Panel B) The proportion of observed individuals from community groupings that had hair mercury content that exceeded the USEPA limit compared to the expected proportion that would exceed the limit based on the proportion of fish caught near (±50 km) the community that exceeded the USEPA limit for water quality. Community groupings are as follows A: communities upstream of mining with low hair mercury (SAL, ITA), B: communities upstream and high hair mercury (BMA), C: communities near mining inputs (SJG, BOA, BOI), D: communities downstream that are downstream of mining (TRE, PPA), and E: communities further downstream (BMD, PAL, PAR, VAL).

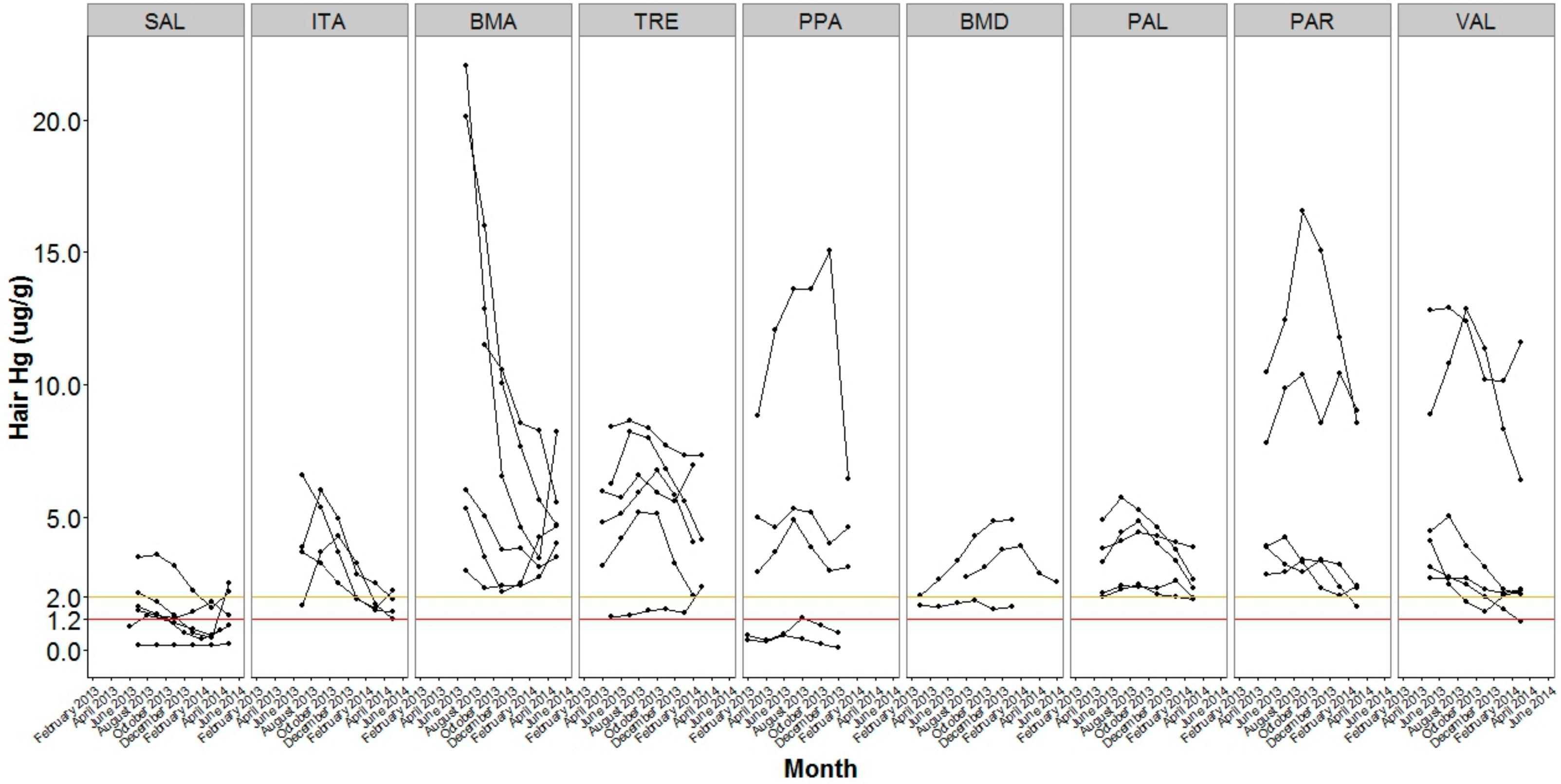

Figure S5. Temporal exposure over a year timeframe was measured in WCBA (n=46). Differences by community are depicted with lines connecting hair mercury contents (ppm) of each individual woman over 2-cm (approximately 2 month) intervals. The red line representing the USEPA limit (1.2 μg/g, dashed red line) and WHO limit (2.0 μg/g, dashed yellow line).

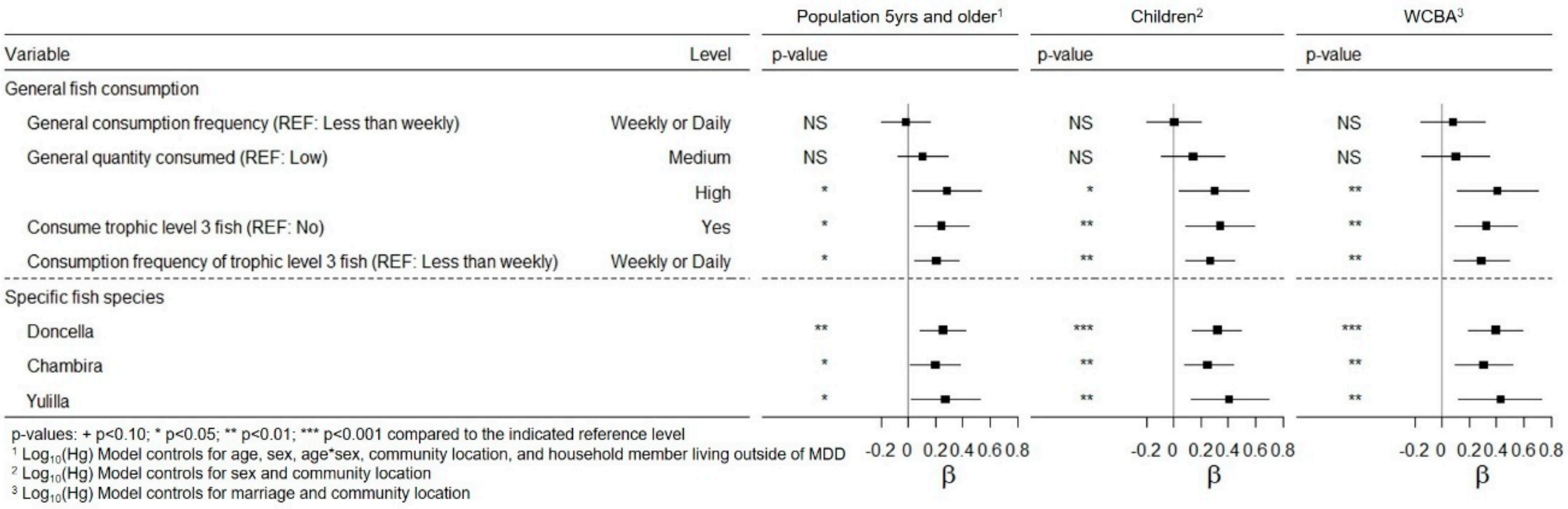

Figure S6. Association between hair mercury and dietary fish consumption in the population five yrs and older, children, and WCBA.

Table S1. Trophic levels of fish.

	Species Name	Trophic level
Ashara	*Leiarius marmoratus*	3
Bagre	*Megalonema platycephalum, Pimelodus sp.*	3
Bocachico	*Prochilodus nigricans*	1
Carachama	*Liposarcus sp., Pterigoplictis disjuntivus, Hypostomus sp., Squaliforma phrixosoma*	1
Chambira	*Hydrolycus pectoralis, Raphiodon vulpinus*	3
Doncella	*Pseudoplatystoma punctifer*	3
Dorado	*Brachyplatystoma rousseauxii*	3
Gamitana		2
Huasaco	*Hoplias malabaricus*	3
Jurel		3
Mojasitas		1
Mota		3
Paco		2
Palometa	*Mylossoma aureum, Mylossoma duriventre*	2
Pana		3
Peje perro		3
Piro	*Megalodoras irwini*	2
Sabalo	*Salminus affinus*	3
Yahuarachi	*Potamorhina altamazonica, Potamorhina latior*	1
Yulilla	*Anodus elongatus*	1
Zungaro Mota		3
Zungaro	*Zungaro zungaro*	3

Table S2. FAO food categories for non-fish items.

Food Category	Food Item
Cereal	Corn
	Kiwicha
	Noodles
	Quinoa
	Rice
White Roots and Tubers	Potatoes
	Yuca
Vegetables	Asparagus
	Beets
	Broccoli
	Corn
	Lettuce
	Onion
	Tomato
Vitamin A Rich Fruits	Mango
	Maracuya (passion fruit)
	Papaya

Other Fruits	Aguaje
	Banana
	Camu camu
	Chirimoya
	Cocona
	Guanabana
	Orange
	Tomato
Organ Meat	Liver
Flesh Meat	Monkey
	Chicken
	Deer
	Grubs
	Guinea pig
	Meat
	Peccary
	Pork
	Reptiles
Eggs	Eggs
Legumes, Nuts, Seeds	Brazil nut*
	Peanut
	Sacha Inchi seed
Milk	Cheese
	Yogurt
Sweets	Chips
	Sweets
Spices, Condiments, Beverages	Aji
	Coffee
	Soda

Table S3. Association between hair mercury and dietary, individual, and household factors in the population 5 yrs of age and older, WCBA, and children.

		Population 5yrs and older[1]			Children[2]			WCBA[3]		
Variable	Level	β	(95% CI)	p-value	β	(95% CI)	p-value	β	(95% CI)	p-value
Fish diet										
Consumption frequency of trophic level 3 fish (REF: Less than weekly)	Weekly or Daily	0.206	(0.046, 0.370)	0.013	0.265	(0.086, 0.445)	0.004	0.286	(0.087, 0.492)	0.006
Non-fish diet										
Cereal consumption frequency (REF: Less than weekly)	Weekly or Daily	-0.224	(-0.457, 0.011)	0.060	-0.237	(-0.473, -0.002)	0.047	-0.604	(-1.237, 0.041)	0.058
Kiwicha		-0.921	(-1.288, -0.558)	< 0.001	-1.269	(-1.907, -0.630)	< 0.001	-0.945	(-1.307, -0.588)	< 0.001
Quinoa		-0.410	(-0.654, -0.171)	0.001	-0.264	(-0.558, 0.030)	0.079	-0.337	(-0.597, -0.072)	0.014
Other fruits consumption frequency (REF: Less than weekly)	Weekly or Daily	-0.287	(-0.613, 0.041)	0.085	-0.397	(-0.692, -0.102)	0.009	-0.182	(-0.633, 0.271)	0.423
Banana		-0.147	(-0.307, 0.013)	0.072	-0.211	(-0.387, -0.040)	0.018	-0.202	(-0.420, 0.018)	0.071
Tomato		-0.150	(-0.309, 0.007)	0.061	-0.231	(-0.370, -0.082)	0.008	-0.187	(-0.395, 0.025)	0.080
Organ meat (liver) consumption frequency (REF: Less than weekly)	Weekly or Daily	-0.305	(-0.546, -0.060)	0.014	-0.374	(-0.676, -0.089)	0.015	-0.364	(-0.665, -0.076)	0.014
Sum of FAO groups consumed weekly or more frequently		-0.035	(-0.077, 0.007)	0.097	-0.026	(-0.062, 0.009)	0.137	-0.034	(-0.134, 0.068)	0.496
Individual										

BMI (REF: normal)	Underweight	-0.179	(-0.341, -0.019)	0.029	-	-	-	-	-	-
	Overweight	-0.020	(-0.114, 0.076)	0.668	-	-	-	0.073	(-0.166, 0.318)	0.542
	Obese	0.083	(-0.032, 0.199)	0.148	-	-	-	0.143	(-0.105, 0.387)	0.246
BMI continuous		0.011	(0.002, 0.020)	0.017	-	-	-	0.015	(-0.007, 0.036)	0.172
Waist circumference		0.004	(-0.000, 0.007)	0.053	-	-	-	0.008	(-0.001, 0.016)	0.084
Hip circumference		0.002	(-0.002, 0.006)	0.369	-	-	-	0.008	(-0.001, 0.017)	0.098
Body fat %		0.001	(-0.003, 0.005)	0.619	-	-	-	0.012	(0.001, 0.024)	0.028
Marriage (REF: Not in a partnership)	In a partnership	0.111	(-0.020, 0.243)	0.084	-	-	-	0.179	(-0.115, 0.467)	0.219
Education (REF: none or less than grade 1)	Grades 1-5	0.101	(-0.054, 0.257)	0.200	-0.038	(-0.169, 0.102)	0.627	0.985	(0.355, 1.611)	0.003
	Grades 6-8	0.166	(0.010, 0.325)	0.038	0.115	(-0.214, 0.479)	0.604	0.887	(0.245, 1.528)	0.007
	Grades 9-12	0.199	(0.026, 0.372)	0.024	-	-	-	1.008	(0.371, 1.645)	0.003
	Technical school/University	-0.005	(-0.229, 0.218)	0.964	-	-	-	0.515	(-0.273, 1.296)	0.153
Time		-	-	-	-	-	-	-0.020	(-0.024, -0.015)	< 0.001

Seasonality (REF: Dry season)	Rainy	-	-	-	-	-	-	-0.141	(-0.180, -0.102)	< 0.001
	Post-rainy	-	-	-	-	-	-	-0.020	(-0.059, 0.019)	0.316
Household										
Average hair Hg of all HH member		-0.161	(-0.206, -0.116)	< 0.001	0.093	(0.061, 0.124)	< 0.001	0.117	(0.064, 0.170)	< 0.001
Father hair Hg		-	-	-	0.073	(0.042, 0.104)	< 0.001	-	-	-
Mother hair Hg		-	-	-	0.056	(0.037, 0.076)	< 0.001	-	-	-
Average parental hair Hg		-	-	-	0.055	(0.035, 0.074)	< 0.001	-	-	-
Spouse hair Hg		0.081	(0.059, 0.104)	< 0.001	-	-	-	0.07457	(0.039, 0.113)	< 0.001

* $p<0.10$; † $p<0.05$; †† $p<0.01$; †††$p<0.001$ compared to the indicated reference level

[1] Log_{10}(Hg) Model controls for age, sex, age*sex, community location, and household member living outside of MDD

[2] Log_{10}(Hg) Model controls for sex and community location

[3] Log_{10}(Hg) Model controls for marriage and community location

Table S4. Association between hair mercury and fish consumption in the population 5 yrs of age and older, WCBA, and children.

		**Population 5yrs and older[1]**			**Children[2]**			**WCBA[3]**		
Variable	Level	β	(95% CI)	p-value	β	(95% CI)	p-value	β	(95% CI)	p-value
General fish consumption										
General consumption frequency (REF: Less than weekly)	Weekly or Daily	-0.017	(-0.195, 0.160)	0.845	0.002	(-0.199, 0.201)	0.987	0.078	(-0.158, 0.313)	0.507

General quantity consumed (REF: Low)	Medium	0.106	(-0.077, 0.287)	0.247	0.141	(-0.091, 0.372)	0.215	0.100	(-0.152, 0.351)	0.431
	High	0.283	(0.031, 0.534)	0.028	0.299	(0.040, 0.553)	0.023	0.406	(0.108, 0.702)	0.009
Consume trophic level 3 fish (REF: No)	Yes	0.243	(0.045, 0.443)	0.018	0.339	(0.089, 0.589)	0.009	0.321	(0.098, 0.547)	0.007
Consumption frequency of trophic level 3 fish (REF: Less than weekly)	Weekly or Daily	0.206	(0.046, 0.370)	0.013	0.265	(0.086, 0.445)	0.004	0.286	(0.087, 0.492)	0.006
Specific fish species										
Doncella		0.253	(0.086, 0.421)	0.004	0.320	(0.135, 0.498)	< 0.001	0.392	(0.193, 0.593)	< 0.001
Chambira		0.197	(0.018, 0.380)	0.032	0.246	(0.083, 0.438)	0.009	0.303	(0.096, 0.517)	0.006
Yulilla		0.271	(0.020, 0.523)	0.032	0.407	(0.131, 0.701)	0.005	0.428	(0.120, 0.732)	0.007

* p<0.10; † p<0.05; †† p<0.01; †††p<0.001 compared to the indicated reference level

[1] Log_{10}(Hg) Model controls for age, sex, age*sex, community location, and household member living outside of MDD

[2] Log_{10}(Hg) Model controls for sex and community location

[3] Log_{10}(Hg) Model controls for marriage and community location

References

1. USEPA, Integrated Risk Information System: Methylmercury (MeHg) (CASRN 22967-92-6). In 2001.
2. USFDA, Title 21 - Food and Drugs - Chapter I Subchapter B - Food for human consumption. In 2016.
